# Synthesis of activity evaluation of flavonoid derivatives as ɑ-glucosidase inhibitors

**DOI:** 10.3389/fchem.2022.1041328

**Published:** 2022-11-15

**Authors:** Hua Zhu, Xin Zhong

**Affiliations:** ^1^ School of Chemistry and Chemical Engineering, Mianyang Teacher’s College, Mianyang, China; ^2^ Dean’s Office, Mianyang Teacher’s College, Mianyang, China

**Keywords:** ɑ-glucosidase, inhibitor, synthesis, flavonoid, screen

## Abstract

Six flavonoid derivatives were synthesized and tested for anti-*α*-glucosidase activities. All derivatives were confirmed using NMR and HRMS and exhibited excellent inhibitory effects on *α*-glucosidase. Derivative **four** exhibited the highest anti-*α*-glucosidase activity (IC_50_: 15.71 ± 0.21 μM). Structure-activity relationship results showed that bromine group would be the most beneficial group to anti-*α*-glucosidase activity. Inhibitory mechnism and inhibition kinetics results showed derivative **four** was a reversible and mixed-type inhibitor. Molecular docking revealed that derivative **four** was tightly bind to the amino acid residues of active pocket of α-glucosidase and formed hydrogen bond, π-π stacking, and Pi-Donor hydrogen with α-glucosidase. Moreover, the physicochemical parameters of all derivatives were assessed using SwissADME software. This results also showed that the hybridization of flavonoid and phenylpropionic acid would be a useful strategy for the development of *α*-glucosidase inhibitors.

## 1 Introduction

**FIGURE 1:**
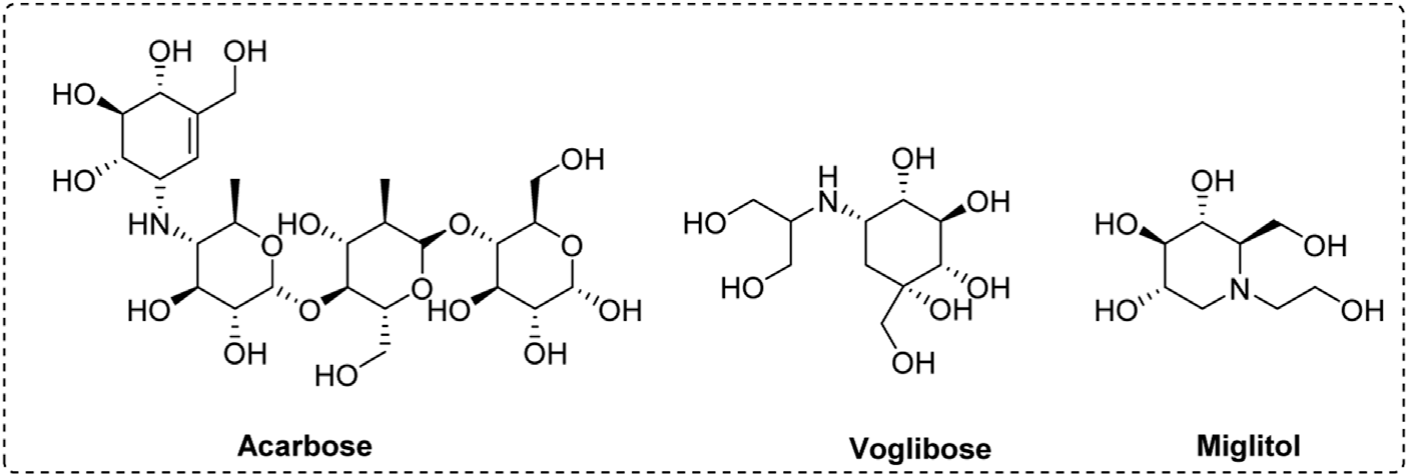
The chemical structure of acarbose, voglibose, and miglitol.

Diabetes mellitus is reported as a common chronic metabolic disorder with hyperglycemia. This hyperglycemia can cause microvascular complications such as cardiovascular, renal, and neurological problems (Forouhi and Wareham, 2014; Proenca et al., 2021). Numerous researches reveal that the hydrolysis of carbohydrates is the major inducement of hyperglycemia (Kokil et al., 2015; Kousaxidis et al., 2020). α-Glucosidase located in the small intestine is one important catalytic hydrolase, which can hydrolyze carbohydrates into absorbable glucose. The excess absorbed glucose causes postprandial hyperglycemia, resulting in diabetes (Ali et al., 2017; Proença et al., 2017; Tsoutsouki et al., 2020). Thence, inhibiting α-glucosidase activity might be an effective strategy for controlling postprandial hyperglycemia (Imran et al., 2015; Rasouli et al., 2017; Sohretoglu et al., 2018). Although lots of α-glucosidase inhibitors have been developed, only a few have been used as clinical drugs for the treatment of diabetes, including acarbose, voglibose, and miglitol (Figure 1). But they are reported to have some adverse reactions during the use (Santos et al., 2018; Hedrington and Davis, 2019). This encourages researchers to find more effective and safety α-glucosidase inhibitors.

Natural products are the important medicinal resources, and many clinical drugs are generated directly or indirectly from natural products (Şőhretoğlu and Sari, 2020; Proenca et al., 2021; Mo et al., 2022). Flavonoids present abundantly in natural products that are a class of compounds with biological and pharmacological activities (Dong et al., 2020; Qin et al., 2020; Wang et al., 2020). Besides, antioxidants, antibacterial, anti-inflammatory, anti-tumor, etc., their anti-α-glucosidase and anti-diabetic properties have gotten more and more attention recently (Amador et al., 2020; Liu et al., 2020; Tang et al., 2020). Now, lots of synthesized and isolated flavonoids were obtained as α-glucosidase inhibitors (Jia et al., 2019; Lu et al., 2020; Oueslati et al., 2020; Zhu et al., 2020).

On the other hand, the esterification modification of natural products is effective strategies to obtain better active compounds or ester prodrugs. Previous works revealed that the esterification modification of coumarin and honokiol produced a series of compounds with better anti-α-glucosidase activity (Sheng et al., 2018; Hu et al., 2021). Thence, in this work, flavonoid skeleton was modified with esterification by substituted phenylpropionic acid, followed by the screening of anti-α-glucosidase activity.

## 2 Results and discussion

### 2.1 Chemistry

**SCHEME 1:**
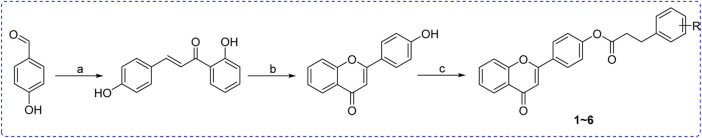
Synthesis of flavonoid derivatives **1**∼**6**. Reagents and condition: **(A)** Piperdine, reflux, 160°C; **(B)** DMSO, I_2_, 100°C; **(C)** Substituted phenylpropionic acid, DMSO, EDCI, DCM, rt.

All flavonoid ester derivatives (**1∼6**) were synthesized according to route outlined in Scheme 1. p-Hydroxybenzaldehyde and 2-Hydroxyacetophenone underwent Claisen-Schmidt condensation to yield 4,2′-dihydroxychalcone, followed by the cyclization reaction to produce 4′-hydroxyflavonoid which reacted with substituted phenylpropionic acid to generate flavonoid derivatives (**1∼6**), respectively. All synthesized flavonoid ester derivatives were identified by ^1^H NMR, ^13^C NMR and HRMS.

### 2.2 α-Glucosidase inhibition assay and SAR analysis

All synthesized six flavonoids were screened for inhibitory activity against α-glucosidase and the results were listed in Table 1. The six flavonoid derivatives existed potential anti-α-glucosidase activity with IC_50_ range of 15.71 ± 0.21–42.06 ± 0.08 μM, which was stronger than that of acarbose (658.26 ± 11.48 μM). Among them, compound **4** showed the strongest inhibitory activity (IC_50_ = 15.71 ± 0.21 μM). The results showed that flavonoid derivatives could be used as potential α-glucosidase inhibitors. That also was said that hybridization of flavonoid skeleton and phenylpropionic acid would be an effective strategy to discover anti-α-glucosidase inhibitors.

**TABLE 1:** The anti-α-glucosidase activity of flavonoid derivatives.

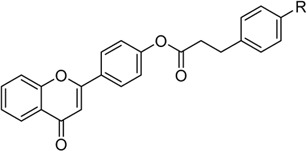		
**Compound**	**R**	**IC_50_ (μM)**
**1**	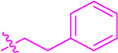	42.06 ± 0.08*
**2**	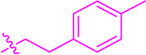	21.55 ± 0.13*
**3**	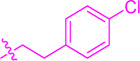	31.09 ± 0.10*
**4**	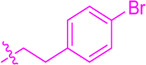	15.71 ± 0.21*
**5**	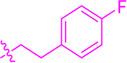	19.66 ± 0.04*
**6**	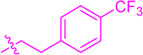	27.54 ± 0.02*
**Acarbose**		658.26 ± 11.48

Compared to acarbose, *p < 0.05.

**FIGURE 2:**
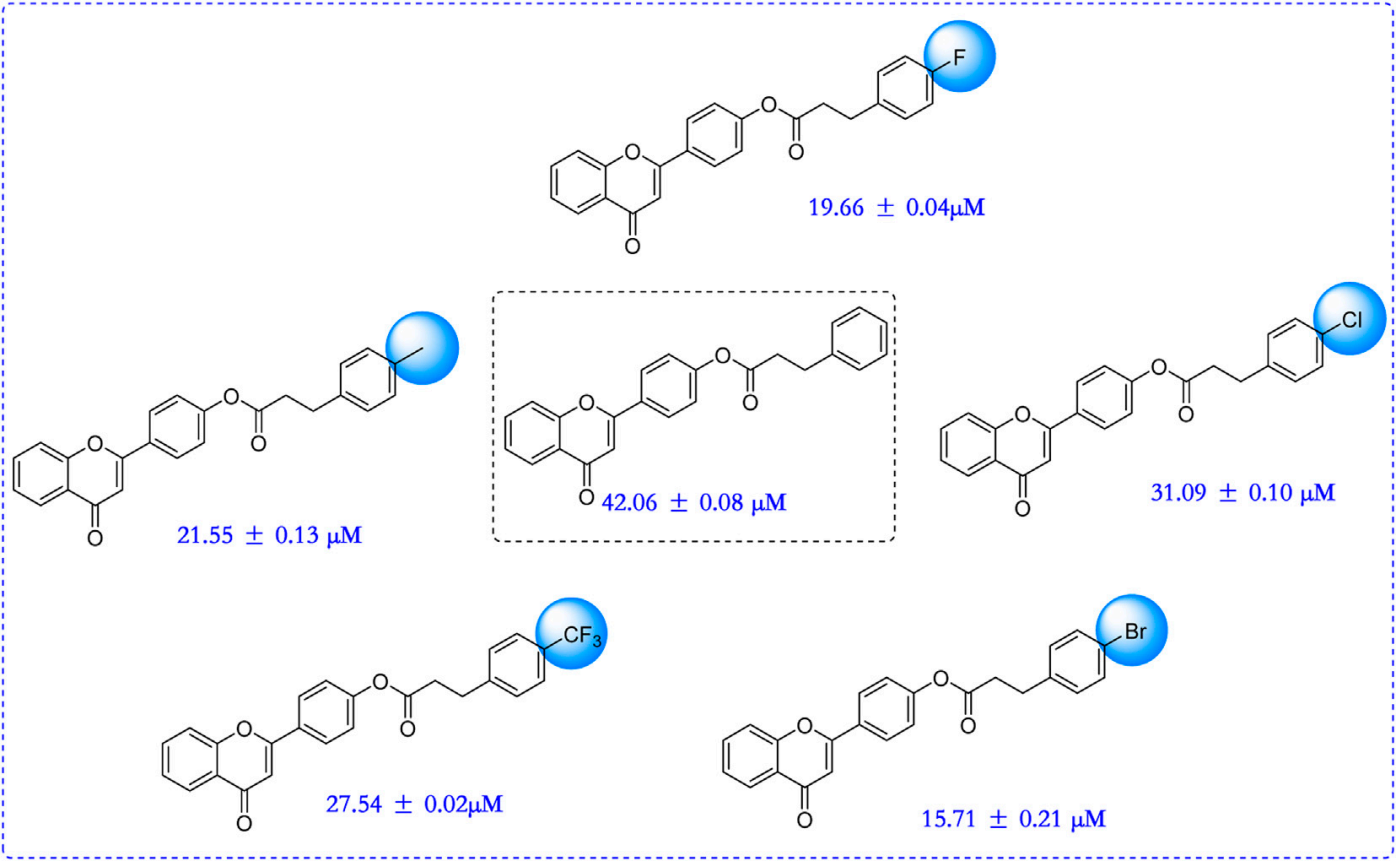
SAR analysis of synthesized six flavonoid derivatives.

In order to better guide future derivatization, the structure-activity relationship (SAR) was analyzed. For all six derivatives, compound **1** with no substituent group at phenylpropionic acid fraction was selected as template molecule, showing an IC_50_ value of 42.06 ± 0.08 μM. Introducing methyl group (compound **2**, IC_50_ = 21.55 ± 0.13 μM), chlorine group (compound **3**, IC_50_ = 31.09 ± 0.10 μM), bromine group (compound **4**, IC_50_ = 15.71 ± 0.21 μM), fluorine group (compound **5**, IC_50_ = 19.66 ± 0.04 μM), and trifluoromethyl group (compound **6**, IC_50_ = 27.54 ± 0.02 μM) on phenylpropionic acid fraction caused effective increase in inhibition activity. Thence, introducing substituents would enhance their anti-α-glucosidase activity and the sequence of substitute group was bromine, fluorine, methyl, trifluoromethyl, chlorine group, and hydrogen. (Figure 2).

### 2.3 Inhibitory mechanism and type assay

**FIGURE 3:**
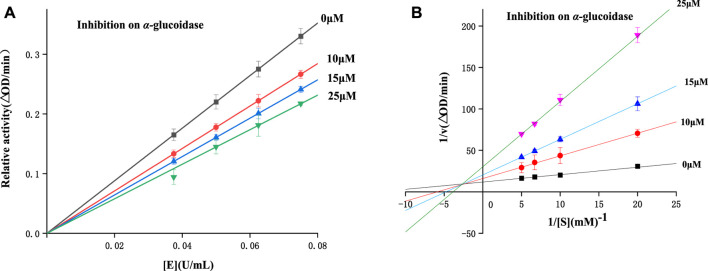
Inhibitory mechanism **(A)** and type assay **(B)** investigation.

To study the inhibitory mechanisms of all derivatives on α-glucosidase, enzyme inhibitory mechnism was detected using compound **4** with the strongest inhibitory. Figure 3A illustrated the plots of enzyme activity vs*.* enzyme concentration. It could be seen that the plots with compound **4** (0–25 μM) all passed the origin, revealing compound **4** as a reversible inhibitor.

The kinetic type was also studied using Lineweaver-Burk plots. As shown in the Lineweaver-Burk plots of enzyme activity vs*.* substrate concentration (Figure 3B), all plots with compound **4** (0–25 μM) intersected at the second quadrant, indicating a mix-type inhibition.

### 2.4 Molecular docking

**FIGURE 4:**
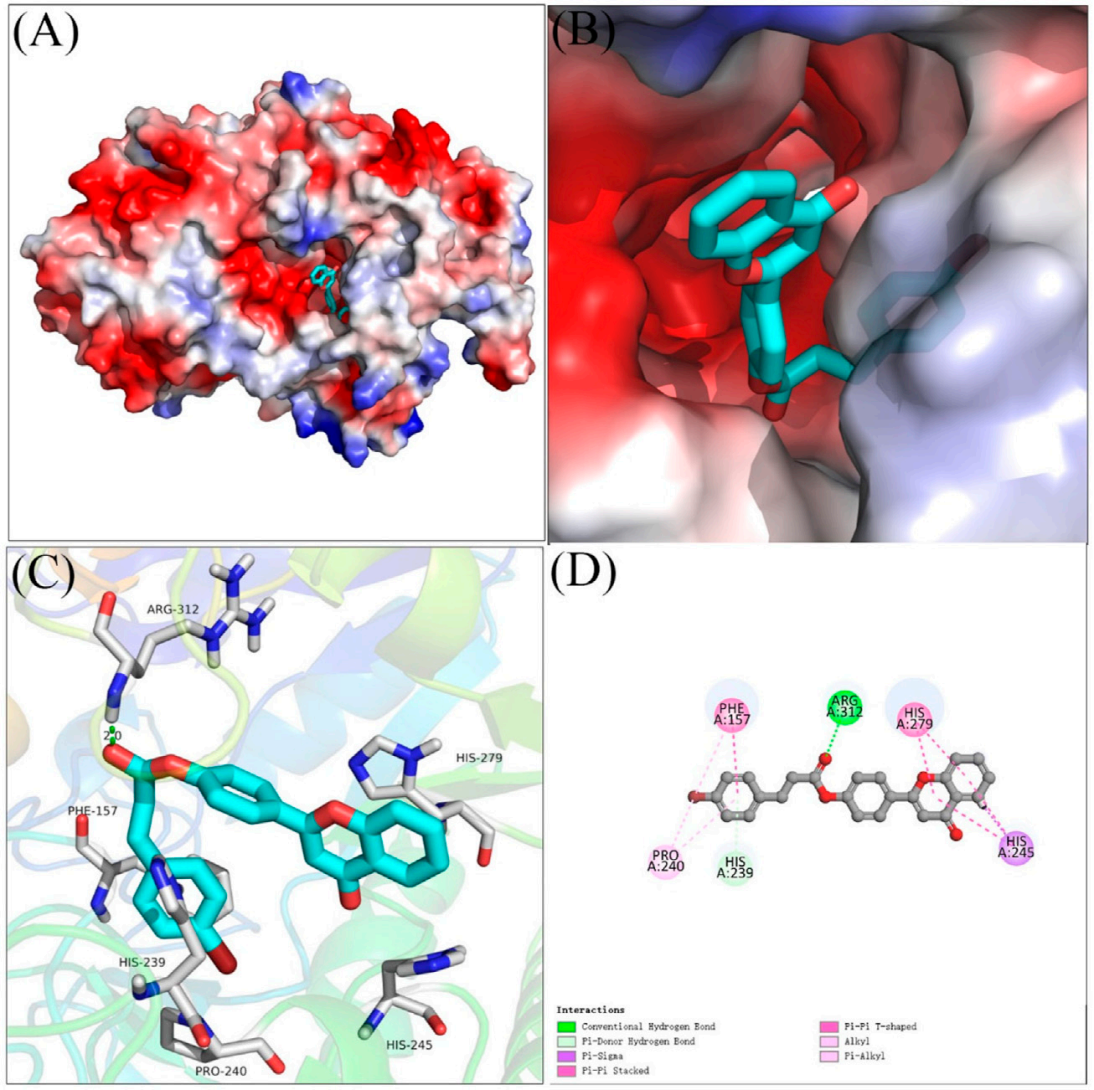
The molecular docking of compound 4 with α-glucosidase. **(A,B)** compound 4 in the active pocket. **(C,D)** detailed binding between compound 4 and enzyem.

Molecular docking of compound **4** with *α*-glucosidase was simulated using SYBYL software, and the binding interactions were analyzed. As shown in Figures 4A–3B, compound **4** bind reliably with the active pocket, the flavonoid section of compound **4** located at entrance of the active pocket, and the bromophenylpropionic acid section located at the interior. Figures 3C,D were the detailed interactions in 3D view and 2D view, respectively. It was seen that the carbonyl moiety formed a hydrogen bond with Arg 312 (2.0 Å), benzene ring of bromophenylpropionic acid section formed a π-π stacking with Phe157 and Pi-Donor hydrogen bond with His239. Moreover, compound **4** also formed hydrophobic interactions with Pro240, His245, and His279.

### 2.5 Physicochemical parameters

The physicochemical parameters of all derivatives were analyzed using SwissADME software. The results showed (Table 2) that all derivatives presented favourable drug-likeness profile. The molecular weight, RB, HBA, HBD, and TPSA of derivatives basically met the standard.

**TABLE 2:** The physicochemical parameters of all derivatives.

Compound	MW (g/mol)	RB	HBA	HBD	TPSA (Å^2^)	LogP_o/w_	WS
**1**	370.4	6	4	0	56.51	4.6	Poorly soluble
**2**	384.42	6	4	0	56.51	4.92	Poorly soluble
**3**	404.84	6	4	0	56.51	5.12	Poorly soluble
**4**	449.29	6	4	0	56.51	5.16	Poorly soluble
**5**	388.39	6	5	0	56.51	4.92	Poorly soluble
**6**	438.4	7	7	0	56.51	5.65	Poorly soluble

## 3 Conclusion

In this study, we synthesized six flavonoid derivatives and tested their anti-*α*-glucosidase activities. All derivatives exhibited excellent inhibitory effects on *α*-glucosidase. Among them, derivative four exhibited the highest anti-*α*-glucosidase activity (IC_50_: 15.71 ± 0.21 μM). Moreover, bromine group was the optimal substituent for activity. Inhibitory mechnism and inhibition kinetics results showed derivative four was a reversible and mixed-type inhibitor. Molecular docking revealed that derivative **four** was tightly bind to the amino acid residues of active pocket of α-glucosidase. Thence, the hybridization of flavonoid and phenylpropionic acid would be a useful strategy for the development of *α*-glucosidase inhibitors. In addition, derivative **four** would be used as a lead compound to develop hypoglycemic drugs.

## 4 Experimental

### 4.1 Materials and methods

*α*-Glucosidase from *Saccharomyces cerevisiae* (EC 3.2.1.20) and *p*-Nitrophenyl-*α*-D-galactopyranoside (*p*-NPG) were purchased from Sigma-Aldrich. All additional reagents and solvents were readily obtained from a commercial source. NMR spectra were obtained on 500 MHz equipment in CDCl_3_. High-resolution mass spectral (HRMS) data was recorded on Apex II using the ESI technique.

### 4.2 General procedure for the synthesis of flavonoid derivatives 1–6

2-hydroxy acetophenone (0.01 M) and appropriate benzaldehydes (0.01 M) were added into piperidine (10 ml) and maintained at 160 C. After reaction was completed, the mixture was treated with ice-cold water and adjusted pH. Then this obtained precipitate was recrystallized in methanol to give 4,2′-dihydroxychalcone. Then, 4,2′-dihydroxychalcone (0.015 M) and iodine (0.015 M) were added into dimethyl sulfoxide, and stirred for 60 min at 140 C. After treated with 20% aqueous sodium thiosulfate, the mixture was extracted with DCM, followed by washing with brine, concentrating in a rotary evaporator, and subsequent recrystallization to obtain 4′-hydroxyflavonoid. 4′-hydroxyflavonoid (0.21 mmol), substituted phenylpropionic acid (0.32 mmol), DMAP (0.42 mmol) and EDCI (0.42 mmol) were added into 10 ml DCM and reacted at room temperature. Then the mixture was quenched by water, extracted with DCM, washed with brine, dried by MgSO_4_, removed solvent under vacuum, and subsequently purified using column chromatography to yield the corresponding flavonoid derivatives **1** ∼ **6**. All ^1^H NMR, ^13^C NMR and HRMS data were summarized into SUPPORTING INFORMATION.

### 4.3 A-glucosidase inhibition and kinetics assay

The α-glucosidase inhibitory activity of compounds (**1 ∼ 6**) was detected as described in previous reports (Adisakwattana et al., 2009; Adisakwattana et al., 2013; Zhang et al., 2022). 10 μl α-glucosidase solution, 10 μl compound were added into 80 μl phosphate buffer, and the mixture was incubated for 10 min. Then, 100 μl *p*-NPG solution was added into the mixture, followed by the absorbance change detection at 405 nm. The inhibition rate (%) = [(OD_1_ - OD_0_)/OD_0_] × 100%, where OD_1_ and OD_0_ were the absorbance of tested compound and blank, respectively. The IC_50_ value was calculated from the plot of inhibition rate vs*.* compound concentration. Acarbose was used as a positive sample. All samples were repeated four times. The enzyme inhibitory mechnism and kinetic type were also determined according to previous reported reports (Adisakwattana et al., 2004; Song et al., 2016).

### 4.4 Molecular docking

Molecular docking of compound **4** with *α*-glucosidase was simulated using SYBYL software according to previous researches (Li et al., 2016; Xu et al., 2019). Compound **4** was constructed and energy minimized using software own programs. The *α*-glucosidase were prepared by hydrogenation and disability rehabilitation. Then, the docking between compound **4** and *α*-glucosidase was operated in the default format.

### 4.5 Statistical analysis

Data was presented as mean ± SD. One-way ANOVA was used to analyze the difference between groups. *p* < 0.05 was considered significant.

## Data Availability

The datasets presented in this study can be found in online repositories. The names of the repository/repositories and accession number(s) can be found in the article/Supplementary Material.
