# Supplementary material for: Synthesis of activity evaluation of flavonoid derivatives as ɑ-glucosidase inhibitors
Source: Front Chem. 2022 Nov 15;10:1041328. doi: 10.3389/fchem.2022.1041328 (PMC9705736; doi:10.3389/fchem.2022.1041328)
Supplement: Supplementary file 1 [file DataSheet1.docx]

Supplementary Material

Synthesis of activity evaluation of flavonoid derivatives as ɑ-glucosidase inhibitors

***General procedure for the synthesis of flavonoid derivatives 1 ~ 6.***

To a solution of 2-hydroxy acetophenone (0.01 M) in piperidine (10 mL), an equimolar quantity of appropriate benzaldehydes was added, then the mixture was maintained at 160 °C. After reaction was completed, the mixture was poured into ice-cold water and adjusted the pH to pH-2 using 6 N hydrochloric acid. Then the obtained yellow precipitate was recrystallized in methanol to give 4,2’-dihydroxychalcone. To the 4,2’-dihydroxychalcone (0.015 M) solution in 50 mL of dimethyl sulfoxide, equimolar amount of iodine (0.015 M) was added, and stirred for 60 min at 140 °C. After treated with 20% aqueous sodium thiosulfate, the mixture was extracted with DCM, followed by washing with brine, concentrating in a rotary evaporator, and subsequent recrystallization to obtain 4’-hydroxyflavonoid. 4’-hydroxyflavonoid (0.21 mmol), substituted phenylpropionic acid (0.32 mmol), DMAP (0.42 mmol) and EDCI (0.42 mmol) were added into 10 ml DCM and reacted at room temperature. Then the mixture was quenched by water, extracted with DCM, washed with brine, dried by MgSO_4_, removed solvent under vacuum, and subsequently purified using column chromatography to yield the corresponding flavonoid derivatives **1** ~ **6**.

***(1, C_24_H_18_O_4_).*** White solid; Yield: 61%; mp: 133 - 134 °C; ^1^H NMR (500 MHz, CDCl_3_) δ 8.24 (dd, *J* = 7.9, 1.7 Hz, 1H), 7.99 - 7.92 (m, 2H), 7.73 (ddd, *J* = 8.7, 7.1, 1.7 Hz, 1H), 7.58 (dd, *J* = 8.6, 1.0 Hz, 1H), 7.45 (ddd, *J* = 8.1, 7.1, 1.1 Hz, 1H), 7.35 (t, *J* = 7.5 Hz, 2H), 7.30 - 7.26 (m, 3H), 7.23 - 7.15 (m, 2H), 6.89 (s, 1H), 3.10 (t, *J* = 7.6 Hz, 2H), 2.94 (t, *J* = 7.7 Hz, 2H). ^13^C NMR (126 MHz, CDCl_3_) δ 178.45, 170.99, 162.94, 156.27, 153.32, 139.88, 134.08, 129.23, 128.70, 128.44, 127.78, 126.62, 125.78, 125.49, 123.68, 122.41, 118.12, 107.37, 36.02, 30.90. HRMS (ESI-MS) m/z: [M + Na]+ calcd for C24H18O4Na: 371.1273; found: 371.1278.

***(2, C_25_H_20_O_4_).*** White solid; Yield: 61%; mp: 135 - 136 °C; ^11^H NMR (500 MHz, CDCl_3_) δ 8.24 (dd, *J* = 7.9, 1.7 Hz, 1H), 7.95 (s, 2H), 7.72 (ddd, *J* = 8.7, 7.1, 1.7 Hz, 1H), 7.61 - 7.55 (m, 1H), 7.48 - 7.41 (m, 1H), 7.24 - 7.12 (m, 6H), 6.87 (d, *J* = 1.5 Hz, 1H), 3.06 (t, *J* = 7.6 Hz, 2H), 2.91 (dd, *J* = 8.0, 6.9 Hz, 2H), 2.35 (s, 3H). ^13^C NMR (126 MHz, CDCl_3_) δ 178.53, 171.17, 162.93, 156.35, 153.40, 136.90, 136.24, 134.11, 129.45, 129.34, 128.40, 127.83, 125.86, 125.54, 123.86, 122.51, 118.22, 107.54, 36.27, 30.57, 21.19. HRMS (ESI-MS) m/z: [M + Na]+ calcd for C25H20O4Na: 385.1430; found: 385.1434.

***(3, C_24_H_17_ClO_4_).*** White solid; Yield: 61%; mp: 112 - 114 °C; ^1^H NMR (500 MHz, CDCl_3_) δ 8.23 (dd, *J* = 7.9, 1.7 Hz, 1H), 7.94 (d, *J* = 8.8 Hz, 2H), 7.71 (ddd, *J* = 8.6, 7.1, 1.7 Hz, 1H), 7.57 (dd, *J* = 8.5, 1.1 Hz, 1H), 7.43 (ddd, *J* = 8.1, 7.1, 1.1 Hz, 1H), 7.33 - 7.28 (m, 2H), 7.23 - 7.16 (m, 4H), 6.83 (s, 1H), 3.06 (t, *J* = 7.5 Hz, 2H), 2.91 (t, *J* = 7.7 Hz, 2H). ^13^C NMR (126 MHz, CDCl_3_) δ 178.50, 170.81, 162.74, 156.35, 153.24, 138.44, 134.05, 132.51, 129.92, 129.51, 128.90, 127.83, 125.87, 125.50, 123.96, 122.50, 122.41, 118.20, 107.66, 35.92, 30.28. HRMS (ESI-MS) m/z: [M + Na]+ calcd for C24H17ClO4Na: 405.0887; found: 405.0888.

***(4, C_24_H_17_BrO_4_).*** White solid; Yield: 61%; mp: 127 - 129 °C; ^1^H NMR (500 MHz, CDCl_3_) δ 8.24 (dd, *J* = 7.9, 1.7 Hz, 1H), 8.00 - 7.92 (m, 2H), 7.73 (ddd, *J* = 8.6, 7.1, 1.7 Hz, 1H), 7.58 (dd, *J* = 8.4, 1.1 Hz, 1H), 7.49 - 7.41 (m, 3H), 7.23 - 7.13 (m, 4H), 6.86 (s, 1H), 3.05 (t, *J* = 7.5 Hz, 2H), 2.92 (t, *J* = 7.5 Hz, 2H). ^13^C NMR (126 MHz, CDCl_3_) δ 178.54, 170.81, 162.83, 156.36, 153.24, 138.96, 134.12, 131.86, 130.33, 129.50, 127.87, 125.88, 125.55, 123.90, 122.44, 120.55, 118.22, 107.63, 35.85, 30.34. HRMS (ESI-MS) m/z: [M + Na]+ calcd for C24H17BrO4Na: 449.0378; found: 449.0383.

***(5, C_24_H_17_FO_4_).*** White solid; Yield: 61%; mp: 103 - 104 °C; ^1^H NMR (500 MHz, CDCl_3_) δ 8.24 (dd, *J* = 8.0, 1.7 Hz, 1H), 7.97 - 7.92 (m, 2H), 7.72 (ddd, *J* = 8.6, 7.1, 1.7 Hz, 1H), 7.58 (dd, *J* = 8.5, 1.0 Hz, 1H), 7.44 (ddd, *J* = 8.1, 7.1, 1.1 Hz, 1H), 7.24 (dd, *J* = 8.5, 5.5 Hz, 2H), 7.21 - 7.15 (m, 2H), 7.06 - 6.98 (m, 2H), 6.87 - 6.83 (m, 1H), 3.07 (t, *J* = 7.5 Hz, 2H), 2.91 (t, *J* = 7.4 Hz, 2H). ^13^C NMR (126 MHz, CDCl_3_) δ 178.53, 170.93, 162.83, 162.73, 160.78, 156.35, 153.27, 135.64, 135.62, 134.11, 130.04, 129.98, 129.45, 127.86, 125.87, 125.54, 123.88, 122.44, 118.21, 115.66, 115.49, 107.59, 36.19, 30.16. HRMS (ESI-MS) m/z: [M + Na]+ calcd for C24H17FO4Na: 389.1180; found: 389.1184.

***(6, C_25_H_17_F_3_O_4_).*** White solid; Yield: 61%; mp: 250 - 251 °C; ^1^H NMR (500 MHz, CDCl_3_) δ 8.24 (dd, *J* = 7.9, 1.6 Hz, 1H), 7.95 (d, *J* = 8.7 Hz, 2H), 7.71 (ddd, *J* = 8.6, 7.2, 1.6 Hz, 1H), 7.58 (dd, *J* = 12.8, 8.1 Hz, 3H), 7.47 - 7.37 (m, 3H), 7.20 (d, *J* = 8.5 Hz, 2H), 6.83 (s, 1H), 3.15 (t, *J* = 7.5 Hz, 2H), 2.96 (t, *J* = 7.5 Hz, 2H). ^13^C NMR (126 MHz, CDCl_3_) δ 178.50, 170.66, 162.71, 156.36, 153.18, 144.09, 134.06, 129.58, 128.93, 127.85, 125.88, 125.78, 125.75, 125.72, 125.69, 125.52, 123.97, 122.38, 118.21, 107.70, 35.62, 30.67. HRMS (ESI-MS) m/z: [M + Na]+ calcd for C25H17F3O4Na: 439.1148; found: 439.1152.

**Compound 1 (**^1^H NMR)

**Compound 1 (**^13^C NMR)

**Compound 2 (**^1^H NMR)

**Compound 2 (**^13^C NMR)

**Compound 3 (**^1^H NMR)

**Compound 3 (**^13^C NMR)

**Compound 4 (**^1^H NMR)

**Compound 4 (**^13^C NMR)

**Compound 5 (**^1^H NMR)

**Compound 5 (**^13^C NMR)

**Compound 6 (**^1^H NMR)

**Compound 6 (**^13^C NMR)
